# All-endoscopic management of benign bone lesions; a case series of 26 cases with minimum of 2 years follow-up

**DOI:** 10.1051/sicotj/2018041

**Published:** 2018-11-22

**Authors:** Hazem A. Farouk, Mostafa Saladin, Wessam Abu Senna, Walid Ebeid

**Affiliations:** Orthopedics & Trauma Surgery Department, Faculty of Medicine, Cairo University, Cairo Egypt

**Keywords:** Bone tumors, Giant cell tumors, Bone cysts, Endoscopic curettage.

## Abstract

*Purpose*: Assessment of the functional and oncologic outcomes regarding endoscopic curettage of different benign bone tumor types within variable anatomic locations.

*Patients and methods*: During the period between February 2012 and December 2016, 26 patients with symptomatic intra-osseous benign bony lesions were included. The age ranged from 3 up to 49 years (mean 20), of 14 females and 12 males. The follow-up duration ranged from 26 up to 58 months (mean 41). Functional scoring was done according to the Revised Musculoskeletal Tumour Society Rating Scale. Anatomic locations of the lesions included: 6 cases in the proximal tibia, 6 cases in the distal femur, 4 cases in the calcaneus, 3 cases in the proximal humerus, 3 cases in the distal tibia, 2 cases in the talus, 1 case in the proximal femur, and 1 case in the distal fibula. The procedure used 4 mm 30° scope for endoscopy, and high speed burrs 3.5–5 mm for extended curettage. Autogenous bone grafting was done in 5 cases, and adjuvant material (polymethylmethacrylate) was needed in 7 cases.

*Results*: After exclusion of one case that was lost in the follow-up, the remaining 25 cases showed full functional recovery at a period of 8–12 weeks, and improved mean functional scores from 20.2 to 28.6/30 post-operatively, with *p* value <0.001 which was considered as a statistically significant result. The oncologic outcome showed 24 cases with adequate healing, while 1 case developed recurrence (aneurysmal bone cyst in the proximal tibia) for which, an open revision surgery was performed. Intra-operative fracture occurred in another case with aneurysmal bone cyst of the proximal femur, which was fixed by flexible nails with complete healing.

*Conclusion*: Endoscopic curettage of different types of intra-osseous benign bony lesions proved to be an effective treatment modality with promising oncologic outcome, improved functional scores, and fast functional recovery.

## Introduction

Benign bone lesions are of various types and may occur in different parts of the skeleton. Some types of benign bone tumors are aggressive in behavior leading to break of their cortical shell and sometimes soft tissue involvement. Thus, management of benign bone tumors differs according to the type and their behavior ranging from non-operative management with observation of the lesion, intra-lesional injection or curettage with or without grafting, or adjuvant therapy. Finally, marginal or even wide resection may be needed in aggressive lesions with soft tissue involvement. All forms of treatment aim at pain relief, promotion of healing, and prevention of adverse complications like recurrence and pathological fractures [1].

Open surgical procedures often cause intraoperative bleeding, prolonged hospitalization, and wound-related complications. So, percutaneous measures have been developed to facilitate the surgery and decrease such undesirable complications. However, these sacrifice the potential advantages of direct exposure needed for adequate surgical management particularly in aggressive lesions with higher risk of recurrence [2–4]. That is why, the use of endoscopy in management of these lesions would be of tremendous help to avoid the problems of open surgery yet, having the advantage of a more accurate assessment of the extent of the lesion with the adequacy of the curettage [5].

The great success of arthroscopic-assisted techniques in management of benign synovial and juxta-articular bone lesions [6–9] aroused the motivation for the development of the new era of endoscopic-aided treatment of benign bone tumors. Thus, we hypothesize that management of benign bone lesions using all endoscopic technique could avoid morbidities associated with open procedures as stated above, yet successfully and efficiently managing the lesions with comparable success rates to open classic techniques. Therefore, the purpose of this study is to evaluate functional and radiological outcomes after all endoscopic treatment of different benign bone lesions.

## Patients and methods

During the period between February 2012 and December 2016, 26 patients with benign bone lesions were enrolled in a prospective case series study to assess the efficacy of endoscopic curettage procedure. The age ranged from 3 up to 49 years with a mean of 18.4 years (SD 11.45), for 14 females and 12 males, with a minimum follow-up duration of 26 months and maximum of 58 months with a mean of 41.1 months (SD 10.2). All Patients with symptomatic benign bone lesions were included in our study. Exclusion criteria included lesions with extra osseous soft tissue extent, and lesions with previous open surgical interventions.

Preoperatively, all patients were clinically and radiologically evaluated and data were recorded. Functional scoring was done according to the Revised Musculoskeletal Tumour Society Rating Scale [10]. This score assesses the pain, restriction to function, and level of satisfaction of the patient either in the upper or lower limbs. Specific assessment to either limbs is incorporated according to the primary role of the limb (for example, gait, Weight bearing and need for external support are items included in assessment of the lower limb valuation while the hand position, ability to lift, and manual dexterity are included in upper limb assessment). Radiological evaluation was done using plain radiographs, while MRI evaluation was needed in cases with doubtful diagnosis. CT-guided core biopsy was required in 15 cases, in which radiography was not conclusive for initial diagnosis.

All operative data were reported including the medications used, type of anesthesia, patient positioning, and the use of tourniquet. The surgical steps included portals choice, the curettage procedure, and tissue material obtained for gross examination and histopathological confirmation. The use of adjuvant materials, grafts, or implants. The need for blood transfusion or drains, the operative time, and finally the overall duration of the hospital stay (Table 1).

**Table 1:** Operative details.

Case	Diagnosis	Anesthesia		Position			Tourniquet		Pump		Portals		Adjuvant Grafts / Implants & Drain	Operative time	Hospital stay
1	Giant cell tumour proximal tibia	Spinal		Supine with knee elevation and bending			Yes		Yes (80 mmhg)		Antero-lateral portal just lateral to the patellar tendon near to the upper end of the lesion Antero-medial portal through the medial surface of the lesion near to its lower end using image intensifier		Hydrogen peroxide Bone cement (PMMA) drain	120 minutes	Three days
**2**	ABC proximal tibia	Spinal		Supine with knee elevation and bending			Yes		Yes (80 mmhg)		Antero-lateral portal just lateral to the patellar tendon near to the upper end of the lesion Antero-medial portal through the medial surface of the lesion near to its lower end using image intensifier		Hydrogen peroxide Bone cement (PMMA) drain	120 minutes	days
**3**	Giant cell tumour distal femur	Spinal		Supine with knee elevation and bending			Yes		Yes (80 mmhg)		Opposing antero-medial and antero- lateral portals on both sides of the upper end of the patella on either sides of the lesion using image intensifier		Hydrogen peroxide Bone cement (PMMA) No drain	90 minutes	Three days
**4**	ABC Calcaneus	Spinal		Lateral with foot elevation and suspension			Yes		Yes (60 mmhg)		Two opposing portals at both medial and lateral surfaces of the calcaneus using image intensifier.		Hydrogen peroxide Bone cement (PMMA) No drain	70 minutes	One day
**5**	UBC distal femur	General		Supine with thigh elevation and knee bending			Yes		Yes (100 mmhg)		Opposing antero-medial and antero- lateral portals on both sides of the upper end of the patella on either sides of the lesion using image intensifier.		No	30 minutes	One day
**6**	Calcaneal lipoma	Spinal		Lateral with foot elevation and suspension			Yes		Yes (60 mmhg)		Two opposing portals at both medial and lateral surfaces of the calcaneus using image intensifier.		Hydrogen peroxide Bone cement (PMMA) No drain	45 minutes	One day
**7**	Calcaneal lipoma	Spinal		Lateral with foot elevation and suspension			Yes		Yes (60 mmhg)		Two opposing portals at both medial and lateral surfaces of the calcaneus using image intensifier.		Hydrogen peroxide Bone cement (PMMA) No drain	40 minutes	One day
**8**	UBC distal tibia	General		Supine with leg elevation			Yes		Yes (60 mmhg)		Anterior portal through the anterior aspect of the lesion near to its upper end Postero-medial portal at the posterior border of medial surface of the lesion near to its lower end using image intensifier.		Drain	45 minutes	One day
**9**	ABC proximal humerus	General Hypotensive measures		Supine semi setting			No		Yes (100 mmhg)		Two portals through the anterior fibers of the deltoid just lateral to the long head of biceps at both upper and lower ends of the lesion using image intensifier.		Drain	90 minutes	Two days
**10**	Cystic fibrous dysplasia proximal tibia	Spinal		Supine with leg elevation and knee bending			Yes		Yes (60 mmhg)		Antero-lateral portal just lateral to the patellar tendon near to the upper end of the lesion Antero-medial portal through the medial surface of the lesion near to its lower end using image intensifier.		Drain	30 minutes	One day
**11**	Chondroblastoma proximal tibia	Spinal		Supine with knee bending and elevation			Yes		Yes (80 mmhg)		Anterior portal medial to the patellar tendon Medial portal through the medial surface of the lesion using image intensifier.		Hydrogen peroxide Autogenous iliac bone grafts No drain	60 minutes	Two days
**12**	UBC distal femur	Spinal		Supine with thigh elevation and knee bending			Yes		Yes (80 mmhg)		Opposing antero-medial and antero- lateral portals on both sides of the upper end of the patella on either sides of the lesion using image intensifier.		Drain	30 minutes	Two days
**13**	UBC distal tibia	General		Supine with leg elevation			Yes		Yes (60 mmhg)		Anterior portal through the anterior aspect of the lesion near to its upper end Postero-medial portal at the posterior border of medial surface of the lesion near to its lower end using image intensifier.		Drain	20 minutes	Two days
**14**	UBC proximal humerus	General Hypotensive measures		Supine semi setting			No		Yes (100 mmhg)		Two portals through the anterior fibers of the deltoid just lateral to the long head of biceps at both upper and lower ends of the lesion using image intensifier.		Drain	40 minutes	Two days
**15**	ABC distal tibia	General		Supine with leg elevation			Yes		Yes (60 mmhg)		Antero-medial portal through the medial surface of the lesion Antero-lateral portal through the lateral surface of the lesion using image intensifier.		Drain	30 minutes	Two days
**16**	ABC proximal tibia	General		Supine with knee elevation			Yes		Yes (80 mmhg)		Antero-medial portal through the medial surface of the lesion near to its upper end Antero-lateral portal through the lateral surface of the lesion near to its lower end using image intensifier.		Above knee cast No drain	30 minutes	One day
**17**	ABC proximal femur	General Hypotensive measures		Supine with hip flexion and thigh elevation			No		Yes (100 mmhg)		Anterior portal through the quadriceps at the anterior border of the lesion near to its upper end. Postero-lateral portal through vastus lateralis near to its lower end using image intensifier.		Two intra-medullary flexible nails No drain	60 minutes	Two days
**18**	Lipoma head of talus	General		Supine with foot elevation			yes		Yes (60 mmhg)		Antero-medial portal of the ankle just below the anterior tip of medial malleolus adjacent to the tibialis anterior tendon Antero-lateral portal of the ankle at sinus tarsi anterior to the peroneal tendons opposing to the other portal using image intensifier.		Autogenous iliac bone grafts No drain	45 minutes	One day
**19**	ABC distal fibula	Spinal		Supine with leg elevation			yes		Yes (80 mmhg)		Two portals direct on the distal fibula One at the upper end of lesion adjacent to its anterior border The other one at the lower end of the lesion adjacent to its posterior border.		No	45 minutes	One day
**20**	Calcaneal lipoma	Spinal		Lateral with foot elevation and suspension			yes		Yes (60 mmhg)		Two opposing portals at both medial and lateral surfaces of the calcaneus using image intensifier.		No	15 minutes	One day
**21**	Chondroblastoma distal femur	Spinal		Supine with knee elevation			yes		Yes (80 mmhg)		Antero-medial portal adjacent to the medial border of the patella near to the upper end of the lesion. Postero-medial portal adjacent to the posterior aspect of the medial femoral condyle near to the lower end of the lesion using image intensifier.		Hydrogen peroxide Autogenous iliac bone grafts No drain	60 minutes	One day
**22**	Giant cell tumour distal femur	Spinal		Supine with knee elevation			yes		Yes (80 mmhg)		Antero-medial portal adjacent to the medial border of the patella near to the upper end of the lesion. Postero-medial portal adjacent to the posterior aspect of the medial femoral condyle near to the lower end of the lesion using image intensifier.		Hydrogen peroxide Bone cement (PMMA) No drain	60 minutes	One day
**23**	UBC proximal humerus	General Hypotensive measures		Supine Semi setting			No		Yes (100 mmhg)		Two portals through the anterior fibers of the deltoid just lateral to the long head of biceps at both upper and lower ends of the lesion using image intensifier.		No	30 minutes	One day
**24**	Non ossifying fibroma proximal tibia	Spinal		Supine with knee elevation			yes		Yes (60 mmhg)		Two portals direct on the medial surface of proximal tibial metaphysis at both upper and lower ends of the lesion using image intensifier.		No	30 minutes	One day
**25**	ABC Body of talus		Spinal		Supine With Foot external rotation	yes		Yes (60 mmhg)		Antero-medial to the ankle just below the anterior tip of the medial malleolus adjacent to the tibialis anterior tendon. Postro-medial to the ankle just anterior to the medial border of the Achilles tendon at the same level opposite to the other portal.		Hydrogen peroxide Autogenous iliac bone grafts No drain		45 minutes	One day
**26**	Chondroblastoma Distal femur		general		Supine With Knee elevation	yes		Yes (60 mmhg)		Single portal direct on medial femoral condyle using image intensifier.		Ethanol Autogenous iliac bone Grafts No drain		45 minutes	One day
															

UBC = unicameral bone cyst, ABC = aneurysmal bone cyst.

The anatomic locations of the lesions included: 6 cases in the proximal tibia, 6 cases in the distal femur, 4 cases in the calcaneus, 3 cases in the proximal humerus, 3 cases in the distal tibia, 2 cases in the talus, 1 case in the proximal femur, and 1 case in the distal fibula.

General anesthesia was performed in 11 cases while regional anesthesia (spinal) was performed in 15 cases. Hypotensive measures were taken to minimize bleeding particularly in cases where tourniquet application was not feasible, also for better field visualization. The duration of hospital stay was 3 days in 3 cases, 2 days in 7 cases, and 1 day in 16 cases. The patient position varied according to the anatomic location of the lesion, whether supine semi-sitting position in proximal humeral lesions. Supine position with hip elevation in lesions of the proximal femur, supine position with elevation and bending of the knee in lesions of the distal femur and proximal tibia, supine position with leg and ankle elevation in lesions of the distal tibia and fibula, and lateral position with heal elevation in lesions of the calcaneus and talus.

Exsanguination and tourniquet application were crucial in our study in order to decrease the blood loss, and for better clear operative field visualization. Tourniquet was applied was applied in 22 cases and not feasible in 3 cases in the proximal humerus and a case in the proximal femur.

Portals choice were dependent on the site of the lesion within the bone, the safe zones to avoid nearby neuro-vascular structures, and the feasibility of proper triangulation to ensure smooth easy movement of instruments within the lesion for adequate visualization and proper curettage of tumor tissues.

**Figure 1:**
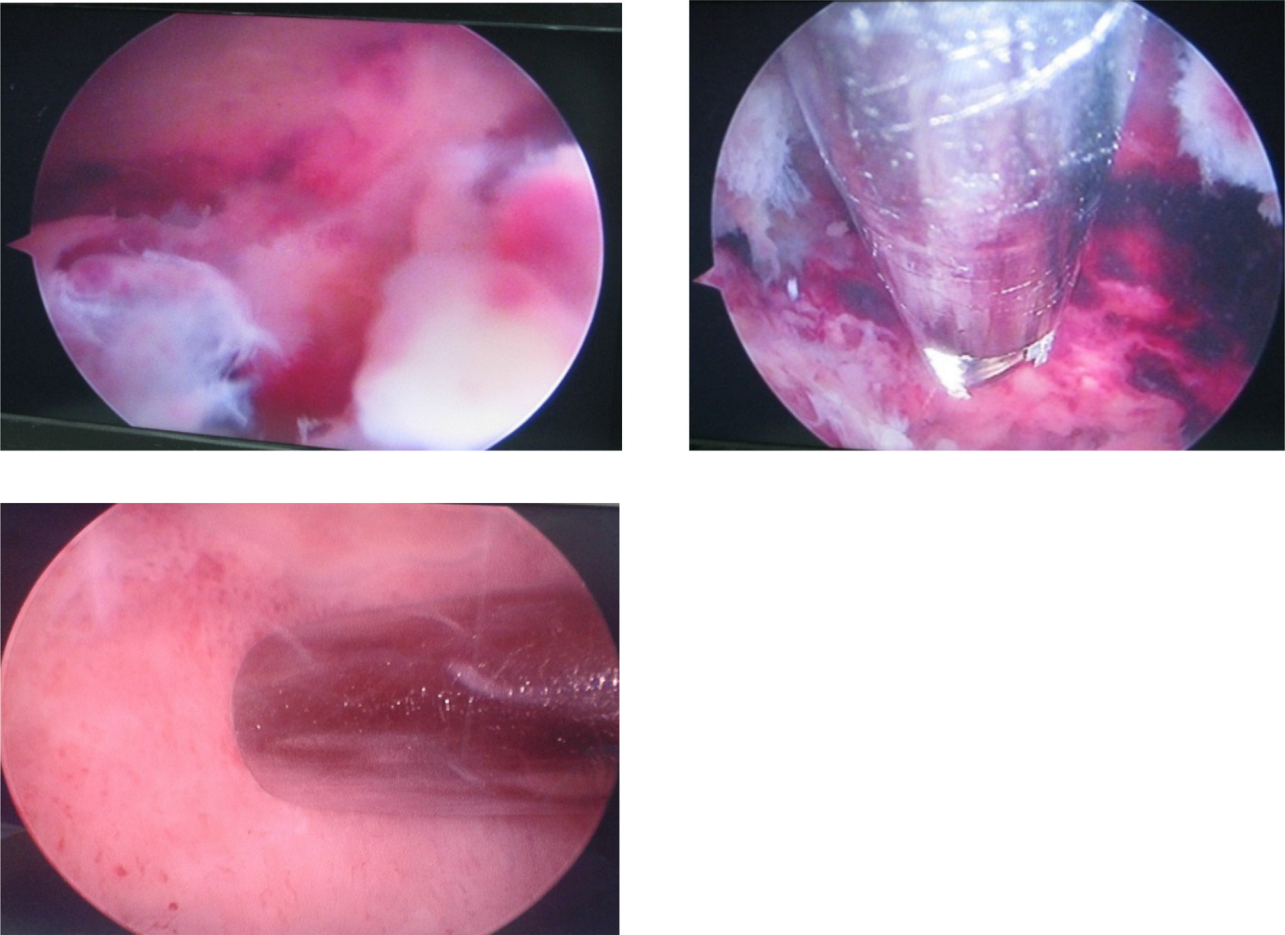
Serial pictures of endoscopic curettage procedure of an ABC (case 7) showing: first pic (top left) the lesion from inside after accessing it through its bony cortex with a trocar. Another portal was created to introduce a 3.5 blade of a motorized shaver followed by a blunt curette (top right). The curettage procedure ends when a healthy bony walls of the lesion (bottom) is clearly visualized from all angles.

Access into the lesions was done using blunt trocars (4 mm in diameter) which were introduced either by T-handle or by pre-drilling the cortex with a 3.5 mm drill bit. None of our lesions were small enough to necessities the use of smaller sized trochars. This was followed by gross curettage using ordinary curved or straight curettes, together with punches and graspers of different sizes. Endoscopic visualization using 4 mm 30° scope was applied. Motorized high speed burrs of different sizes ranging from 3.5 to 5 mm were used for extended curettage (Figure 1).

Autogenous bone grafting was done in 5 cases. While adjuvant material (polymethylmethacrylate) was needed in 7 cases by injection through any of the portals.

The patients were followed up at 2 weeks for stitches removal, then at 6 and 12 weeks and every 3 months afterwards till the end of first year, then half yearly afterwards. Functional evaluation using the same rating scale was done; also the time of return to normal functional activities was reported. Also radiographic evaluation of healing or progression of lesions was done.

## Results

The final functional scoring results according to Revised Musculoskeletal Society Rating Scale were score 30 in 13 cases, score 29 in 3 cases, score 28 in 5 cases, score 27 in 2 cases, score 26 in 1case, score 20 in 1 case, and 1 case was lost follow-up with the mean score (28.6).

**Figure 2:**
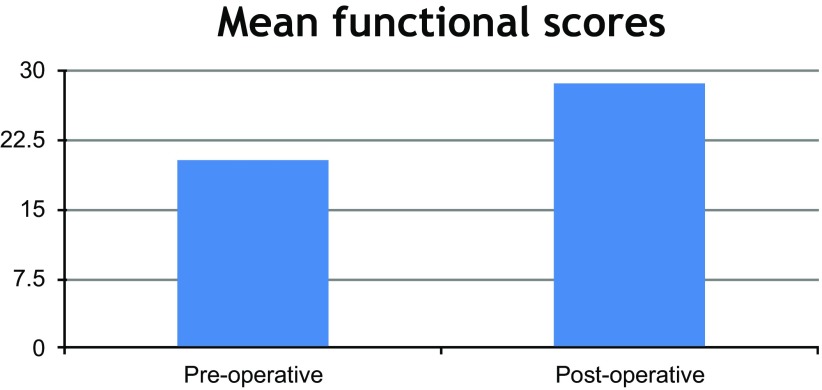
A column chart demonstrating the statistical analysis of the functional scoring results.

Statistical analysis of the functional scoring results after excluding the missed follow-up case using paired T-Test described in (Figure 2) as the following.

The pre-operative mean functional score was (20.2) with 1.89 standard deviation. The post-operative mean functional score was (28.6) with 2.25 standard deviation with *p* value <0.001which was considered as a statistically significant result.

One case with calcaneal lipoma had been lost in the follow-up, and the remaining 25 cases were followed up for the time they return to full function which ranged from 8 up to 12 weeks.

**Figure 3:**
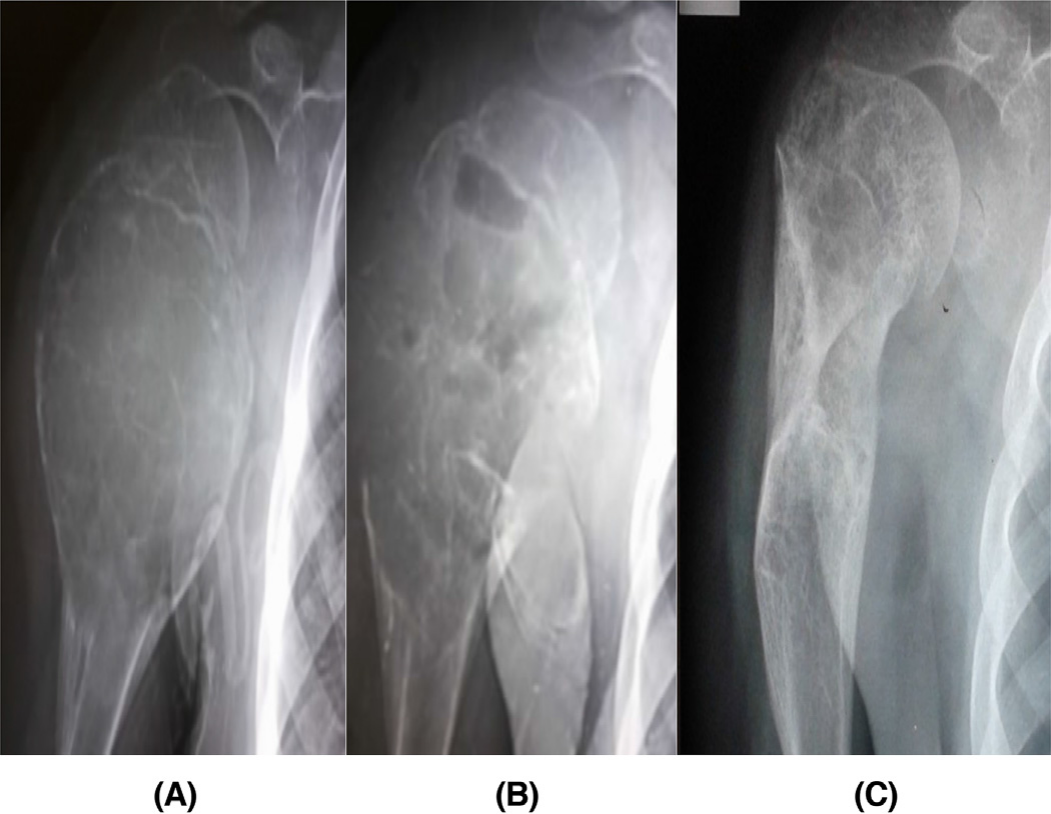
Serial plain radiographs (A) 11 years old patient with ABC lesion in the proximal humerus (case 9), (B) 4 weeks follow-up after endoscopic curettage without grafting showed new bone formation, and (C) complete healing and remodeling after 24 months.

**Figure 4:**
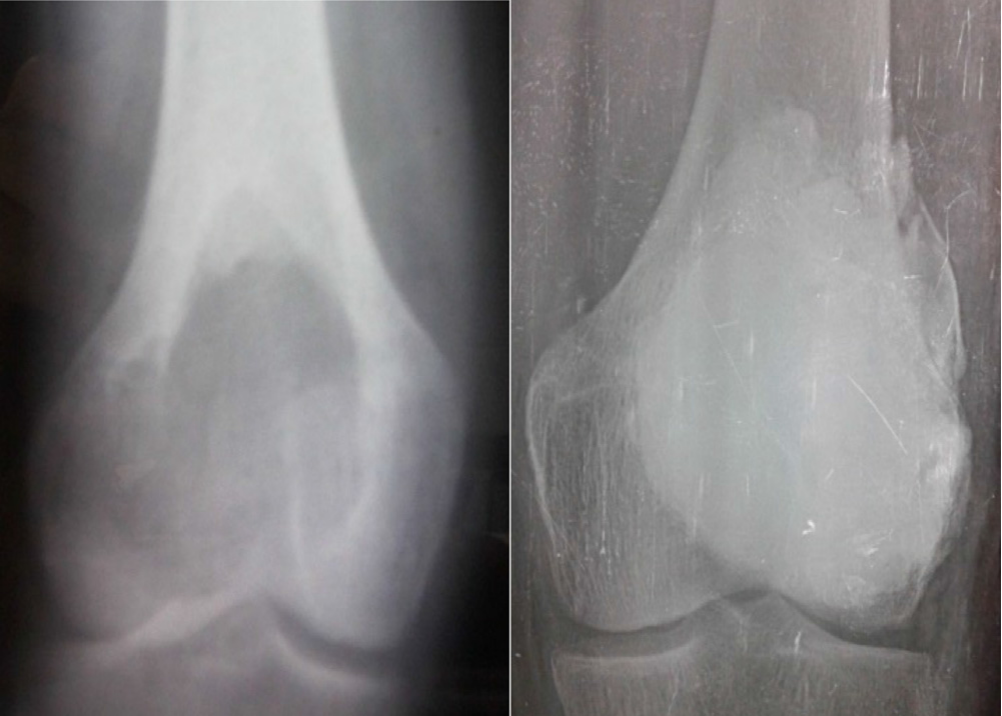
Serial plain radiographs (A) 18 years old patient with GCT in the distal femur (case 3), (B) 30 months last follow-up after endoscopic curettage and percutaneous bone cement application with no radiologic evidence of recurrence.

**Figure 5:**
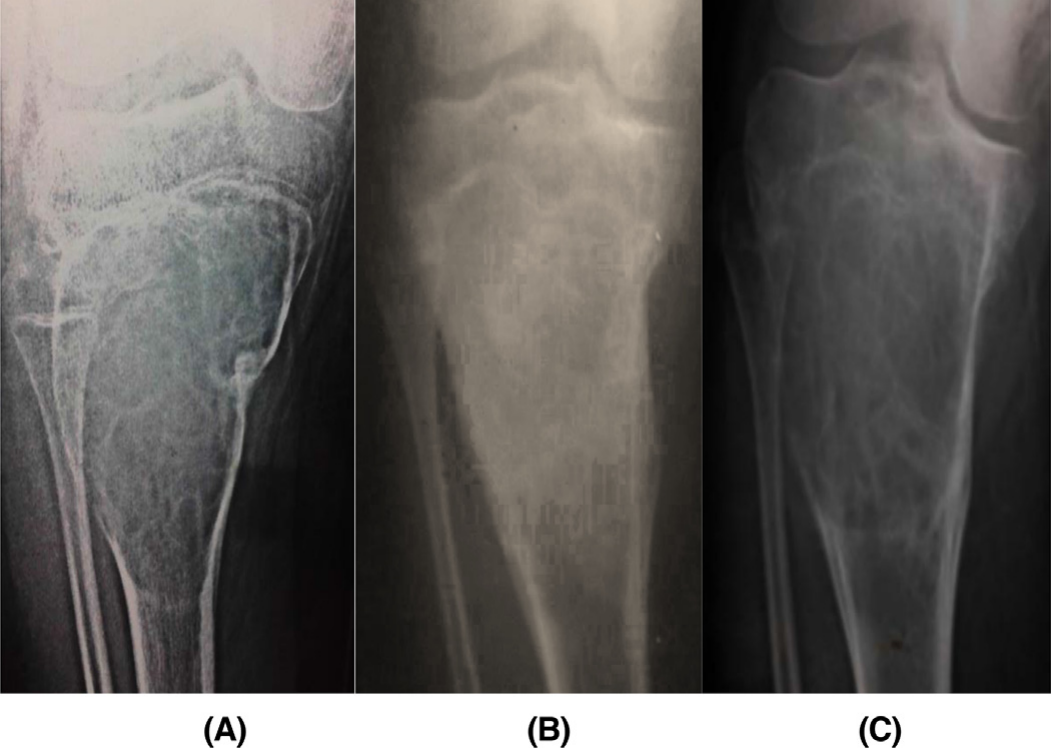
Serial radiographs of (A) 11 years old patient with ABC in the proximal tibia (case 16), (B) 3 months follow-up showed proper healing and cortical thickening, and (C) 5 months follow-up showed lysis in previously healed areas and bone expansion (signs of recurrence).

The radiological outcome of the lesions showed 24 cases with adequate healing, while 1 case developed recurrence (ABC in the proximal tibia), and 1 case (Calcaneal Lipoma) had lost follow-up (Figures 3–5).

The final post-operative histopathologic diagnosis was as following: 8 cases of aneurysmal bone cyst, 6 cases of unicameral bone cyst, 4 cases of intra-osseous lipoma, 3 cases of giant cell tumour, 3 cases of chondroblastoma, a case of fibrous dysplasia, and finally a case of non-ossifying fibroma (Table 2).

**Table 2:** A comparison of different clinical trials to the current study.

Study	Number of cases	Anatomical sites	Pathological types	Functional result	Oncological result	Complications
Stricker ^11^1995	3	Femoral head	Chondroblastoma	Good	Healed	.........
Bonnel et al. ^12^1999	1	Calcaneus	UBC	Good	Healed	.........
Otsuka et al. ^13^2001	4	Patella Proximal humerus Proximal tibia Calcaneus	Atypical ABC no aneurysmal dilatation	Good	Healed	.........
Otsuka et al. ^14^2002	1	Calcaneus	Chondroblastoma	Good	Healed	.........
Dietz et al. 2007^15^	2	Hand	Enchondroma	Good	Healed	.........
Yildirim et al. ^16^2010	1	Calcaneus	UBC	Good	Healed	.........
Innami et al. ^17^2011	13	Calcaneus	UBC	Good	Healed	.........
Yildirim et al. ^18^2011	13	Calcaneus	UBC	Good	Healed	.........
Choi et al. ^5^2014	32	Not included	9 UBC 6 Fibrous dysplasia 5 Enchondroma 4 NOF 3 Bone infarcts 1 ABC 1 Chondroblastoma 1 Osteoblastoma 1 Lipoma 1 Brodie abscess	Not included	21 Excellent 6 Good 1 Poor 4 Recurrence	4 Recurrence 1 Fracture 1 Infection
Current study	26	6 Proximal tibia 6 Distal femur 4 Calcaneus 3 Proximal humerus 3 Distal tibia 1 Proximal femur 1 Fibula 2 Talus	8 ABC 6 UBC 4 Lipoma 3 GCT 3 Chondroblastoma 1 Fibrous dysplasia 1 NOF	Except cases(7,16) All cases reached full recovery within 8 to12 weeks	24 Healed 1 Recurrence 1 Lost follow-up	1 Recurrence and Varus lower limb deformity 1 Fracture 1 Lost follow-up

UBC = unicameral bone cyst, ABC = aneurysmal bone cyst, NOF = non-ossifying fibroma, GCT = giant cell tumour, BFH = benign fibrous histocytosis.

Complications in our study included one case of local recurrence that was associated with lower limb varus malalignment. Another complication encountered was intra-operative fracture during the endoscopic procedure in one case. No reported scope related complications including: Portal track infection, fluid leakage with compartment syndrome, and thromboembolic complications. No reported neuro-vascular injury.

**Figure 6:**
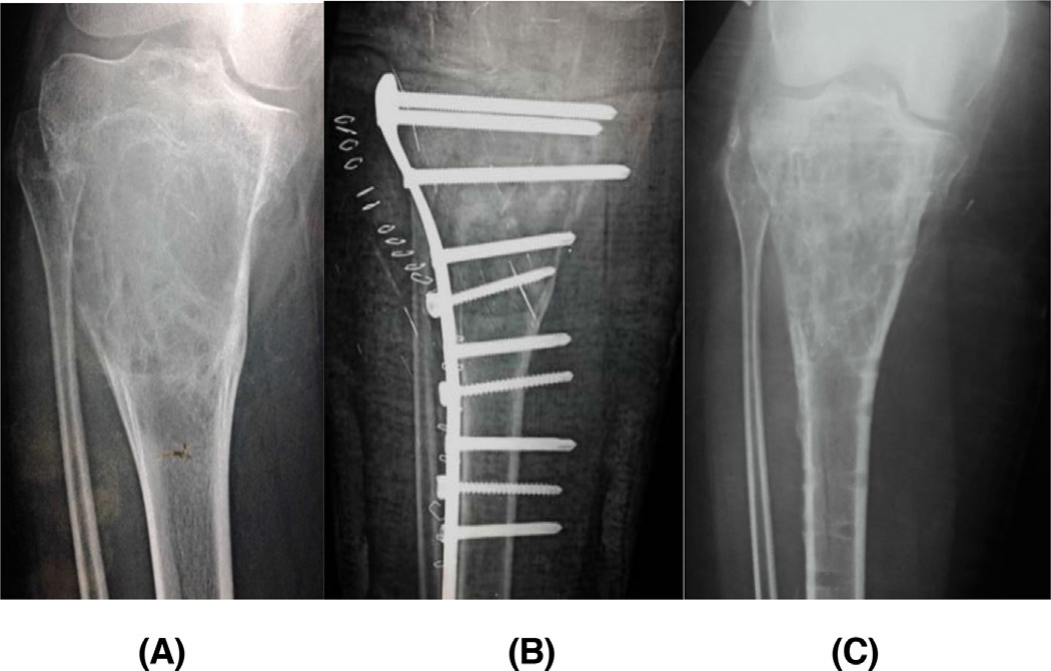
(A) Plain radiograph showed varus mal-alignment of recurrent ABC in the proximal tibia (case 16), (B) post-operative radiograph after revision surgery, and (c) last follow-up radiograph 32 months post-operative showed complete healing.

Local recurrence developed in 11 years old female patient with an expansile aneurysmal bone cyst in the proximal tibia (case 16). Endoscopic curettage without grafting was carried out. Follow-up serial radiographs showed proper healing and cortical thickening, so assisted weight bearing started at 8 weeks, and full weight bearing at 10 weeks post-operatively. At a period of 4 months she experienced gradual progressive pain which was aggravated by weight bearing, and a concomitant night pain. Also she developed an associated lower limb varus malalignment. The follow-up plain radiograph showed lysis in previously healed areas with cortical expansion (signs of recurrence) as shown previously (Figure 5). Further MRI study and CT-guided core biopsy had confirmed the diagnosis of recurrence. Finally open extended curettage with iliac crest grafting and internal fixation using plate and screws was done (Figure 6). The revision surgery operative time was 120 minutes, blood loss was around 1500 mL where 2 units of blood were given, and the overall duration of hospital stay was 4 days.

Another case encountered an intra-operative fracture in 10 years old female patient with an aneurysmal bone cyst in the proximal femur (case 17). Endoscopic curettage was carried out without grafting, together with intra-operative fixation of the fracture by intramedullary flexible nails. Then she was splinted in hip spica cast for 8 weeks till the start of healing and union. After which full weight bearing was allowed at time of nails removal (12 weeks). Full bony union and complete healing of the lesion were achieved.

## Discussion

This pilot study serves to throw more light on the results of using endoscopy in different benign bone tumors. The question was could we do safe endoscopic treatment for different benign bone lesion types in variable anatomical locations? Adequacy was assessed through evaluating the oncological and functional outcome. Safety of the procedure was assessed by evaluating the associated complications. Most of the reported clinical trials in the literature showed successful oncological results (Table 3). The only exception was Choi et al. [5] who reported 4 cases of recurrences (simple bone cyst, fibrous dysplasia, aneurysmal bone cyst, and osteoblastoma) out of 32 cases. Endoscopic surgical treatment maintains the structural integrity and the periosteal sleeve of the affected bones, with minimal cortical breakdown by small sized portals, so faster bone healing could be achieved. It has a minimal risk for local soft tissue injury and hence faster healing and better functional recovery. None of the related clinical trials in the literature up to our knowledge used a scoring system for functional evaluation of their cases and did not measure the time needed for recovery. Moreover, in the current study apart from the patient lost to follow up and the patient who developed local recurrence, all patients reached normal full functional activities at a period of 8–12 weeks post-operatively.

**Table 3:** Summary of the study results.

Case	Age, Sex	Core-biopsy result	Definitive diagnosis	Procedure	Follow-up period	Functional scoring Pre\Post	Radiologic results	Complications
1	18 F	ABC	GCT of proximal tibia	Endo curettage and cement	58 months	20 28	Healed	.............
2	36 F	ABC	ABC of proximal tibia	Endo curettage and cement	58 months	18 27	Healed	.............
3	18 F	GCT	GCT of distal femur	Endo curettage and cement	57 months	20 28	Healed	.............
4	21 M	ABC	ABC calcaneus	Endo curettage and cement	54months	18 28	Healed	.............
5	6 M	Inconclusive	UBC distal femur	Endo curettage No graft	54 months	22 30	Healed	.............
6	45 M	––––	Calcaneal lipoma	Endo curettage and cement	52 months	24 27	Healed	–––––––-
7	37 F	––––––-	Calcaneal lipoma	Endo curettage and cement	50 months	22 –––-	Lost	Lost follow-up
8	3 F	Inconclusive	UBC distal tibia	Endo curettage No graft, slab	49months	19 30	Healed	.............
9	11 F	ABC	ABC proximal humerus	Endo curettage No graft	49 months	20 28	Healed	.............
10	16 M	––––-	F.D proximal tibia	Endo curettage No graft	45 months	22 30	Healed	.............
11	19 M	Chondroblastoma	Chondroblastoma Proximal tibia	Endo curettage and grafting	45 months	19 30	Healed	.............
12	23 F	––––	UBC distal femur	Endo curettage No graft	39 months	22 30	Healed	.............
13	14 F	––––-	UBC distal tibia	Endo curettage No graft	36 months	20 28	Healed	.............
14	15 M	––––	UBC proximal humerus	Endo curettage No graft	36 months	22 30	Healed	.............
15	9 F	––––-	ABC distal tibia	Endo curettage No graft	35 months	19 30	Healed	.............
16	11 F	UBC	ABC proximal tibia	Endo curettage No graft, slab	35 months	16 20	Recurrent	Recurrence Varus deformity
17	10 F	ABC	ABC proximal femur	Endo curettage No graft, I.M nails spica	35 months	18 29	Healed	Fracture
18	11 M	Lipoma	Lipoma head of talus	Endo curettage and graft, slab	34 months	22 30	Healed	.............
19	43 F	ABC	ABC distal fibula	Endo curettage No graft	34 months	20 29	Healed	.............
20	49 M	–––––	Calcaneal lipoma	Endo curettage No graft	33 months	22 26	Healed	.............
21	19 M	Chondroblastoma	Chondroblastoma distal femur	Endo curettage and graft	32 months	20 30	Healed	.............
22	22 F	GCT	GCT distal femur	Endo curettage and cement	32 months	18 30	Healed	.............
23	12 F	–––––-	UBC proximal humerus	Endo curettage No graft	32 months	22 30	Healed	.............
24	16 M	NOF	NOF proximal tibia	Endo curettage No graft	31 months	20 30	Healed	.............
25	23 M	……………….	ABC body Talus	Endo curettage And graft	28 months	22 29	Healed	……………
26	12 M	……………	Chondroblastoma distal femur	Endo curettage And graft	26 months	22 30	Healed	…………...

M = male, F = female, Endo = endoscopic, I.M = intramedullary, F.D = fibrous dysplasia, UBC = unicameral bone cyst, ABC = aneurysmal bone cyst, NOF = non-ossifying fibroma, GCT = giant cell tumour.

To our knowledge this study is considered as the second largest clinical trial after Choi et al. [5] in endoscopic management of benign bone tumors; regarding the number of cases included, and the variability of the anatomical locations and the pathological types of the treated lesions. Also the current study would be considered as the first clinical trial in endoscopic management of giant cell tumor of the bone (cases 1, 3, 22).

Complications in our study included 2 cases only; a case of local recurrence with lower limb varus malalignment, and a case with intra-operative fracture. This is compared to Choi et al. [5] study which is the only study to report complications.

Comparison of the study results to other reported common clinical trials in the literature is described in (Table 3).

Our experience at the end of the study would suggest that variable pathologic types of contained intraosseous benign bone lesions within variable anatomical sites could be managed safely and effectively by endoscopic techniques. However technical difficulties varied from one lesion to another according to different variables. First, less visual field clarity was found with lesions of the proximal humerus (cases 9, 14, 23), and the proximal femur (case 17). This was related to lack of tourniquet application, so we recommend hypotensive anesthesia, proper patient positioning, and using a pump system. Second, considerable difficulty in portals planning for the proximal femur lesion (case 17) was noted due to narrow safe zone, which made the process of triangulation more difficult. Thus, excessive manipulation (torsional stresses) was necessary during surgery, which leaded to an intraoperative fracture. Third, endoscopic management would be considered as the option of choice in lesions of the distal tibia and the foot as they have poor soft tissue envelope with more risk of open wound-related complications. Thus, it would be associated with lower morbidity and better outcome. Fourth, benign bone lesions with solid components (such as giant cell tumor and chondroblastoma), and cystic lesions with abundant tissue and high vascularity (aneurysmal bone cyst) were more technically demanding, regarding a longer operative time needed for percutaneous gross tissue removal. Lastly, no major variation was found regarding the size of different bone lesions. However, massive lesion would be expected to take longer operative time. Also small-sized lesions would need an additional equipment set (scopes 2.5 or even 1.9 mm) to deal with properly.

The weaknesses of the current study include lack of comparison to other conventional operative techniques due to the attenuated sample size. Moreover, we could not compare the results of endoscopic treatment of different types of benign bone tumors as this would need larger-sized patient groups for each type. Lastly, we could claim that the follow-up period was not long.

Our future recommendation is to use larger sample size and a longer follow-up period. Randomized control clinical trials are needed to compare the results of endoscopic surgery to that of other conventional types of treatment modalities. Also more trials will be needed to compare the effectiveness of endoscopic surgery within different groups of benign bone lesion types in variable anatomical locations.

## Conclusion

Endoscopic-assisted surgical technique proves to be an effective method in management of symptomatic cases with contained intra-osseous benign bony lesions of different pathologic types in different anatomic locations. Also as being a percutaneous minimally invasive technique, it has the potential advantages of keeping the structural integrity and the periosteal continuity of the affected bone, with better healing potentials and faster recovery. Finally it provides proper direct exposure of the entire lesion with careful assessment of the adequacy of curettage, and thus a lower risk of recurrence.

## Conflict of interest

The authors declare no conflict of interest.
